# Crystal structures of the A_2A_ adenosine receptor and their use in medicinal chemistry

**DOI:** 10.1186/2193-9616-1-22

**Published:** 2013-12-20

**Authors:** Kenneth A Jacobson

**Affiliations:** Molecular Recognition Section, Laboratory of Bioorganic Chemistry, NIDDK, National Institutes of Health, Bethesda, Maryland 20892-0810 USA

**Keywords:** G protein-coupled receptors, Adenosine receptors, Crystal structures, Modeling, Computer-aided drug discovery

## Abstract

New insights into drug design are derived from the X-ray crystallographic structures of G protein-coupled receptors (GPCRs), and the adenosine receptors (ARs) are at the forefront of this effort. The 3D knowledge of receptor binding and activation promises to enable drug discovery for GPCRs in general, and specifically for the ARs. The predictability of modeling based on the X-ray structures of the A_2A_AR has been well demonstrated in the identification, design and modification of both known and novel AR agonists and antagonists. It is expected that structure-based design of drugs acting through ARs will provide new avenues to clinically useful agents.

## Purpose

A large fraction of currently marketed pharmaceuticals act via G protein-coupled receptors (GPCRs), and understanding recognition and function of GPCRs at a precise molecular level is essential for future drug discovery (Jacobson & Costanzi 2012). Action of a drug via a GPCR is either through direct binding to the receptor (as orthosteric agonist or antagonist or as allosteric modulator) or by affecting the levels of the endogenous transmitter that are available to activate it. Among the many naturally-occurring extracellular signaling molecules, nucleosides and nucleotides serve to both modulate biological processes in many organs and tissues and to maintain homeostasis. In addition to their many intracellular functions and ion channel activation, these ubiquitous signaling molecules act outside the cell through GPCRs. These receptors include eight subtypes of P2Y receptors (P2YRs), which are all activated by nucleotides but not nucleosides, and four subtypes of adenosine receptors (ARs). The increased release and production of extracellular nucleotides and adenosine, which are localized rather than systemic, are typically a direct function of stress to an organ or tissue, such as hypoxia (Chen et al. 2013). The resulting elevated level of endogenous agonist increases a tonic activation of one or more of these receptors to produce a protective response. In some cases, such as the adenosine “halo” surrounding tumor cells (Antonioli et al. 2013), the protective response is undesired clinically, and in which case an antagonist might be more applicable therapeutically. Nucleotide action through P2YRs tends to boost the innate immune system, i.e. the immediate defensive response.

There is renewed interest in developing AR or P2YR modulators for therapeutic use, due to recent advances in understanding the related biological processes and increased structural knowledge of the receptors. We and other labs are exploring structure-activity relationships (SAR) at ARs and P2YRs, in order to synthesize and characterize selective agents as pharmacological probes and as potential therapeutic agents. We emphasize four aspects of studying these receptors: 1) design and synthesis of novel and selective agonists and antagonists to expand the SAR; 2) structure-function studies of the receptor proteins; 3) exploration of the novel biological role of such receptors; and 4) conceptualization of future therapeutics. In this effort, there is a tight coupling of organic synthetic methodology, molecular modeling, structural biology, and pharmacology.

## Main text and discussion

We are designing more potent and subtype-selective receptor ligands, based on an understanding of the molecular recognition in the binding site. New insights into drug design are derived from the X-ray crystallographic structures of GPCRs (Jacobson & Costanzi 2012), and ARs are at the forefront of this effort (Jaakola et al. 2008;Dore et al. 2011;Xu et al. 2011). Although few compounds that act at these two receptor families are approved for clinical use, the ARs serve as important test cases for structure-based ligand design for GPCRs in general. Since 2008, the availability of X-ray crystallographic structures of the human A_2A_AR in complex with xanthine and non-xanthine antagonists (Jaakola et al. 2008;Dore et al. 2011), has allowed more precise molecular modeling of the 3 dimensional structures of the A_2A_AR and related receptors with other bound ligands. Recently, a very high resolution structure of an antagonist-bound A_2A_AR (1.8 Å) was reported and shown to predict allosteric interactions at a site deeper than the orthosteric ligand binding region (Liu et al. 2013;Gutiérrez-de-Terán et al. 2013). The accuracy of many previous AR modeling and docking studies (Ivanov et al. 2009) is a fortuitous result of the close structural similarity of ARs and rhodopsin, the GPCR used for nearly a decade as the most closely related protein template for modeling Family A GPCRs.

**Figure 1:**
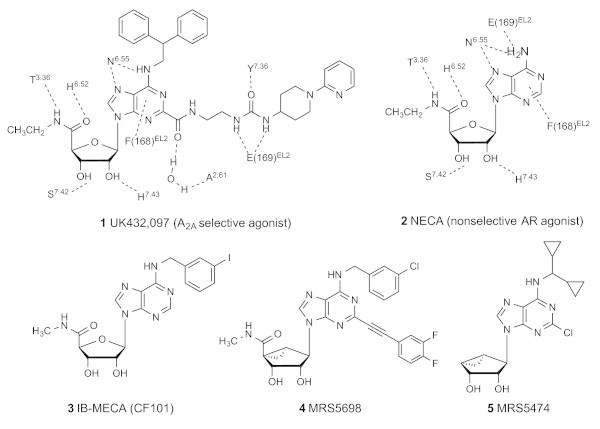
**Two of the AR agonists that were co-crystallized with the human A**_**2A**_ **AR (1, 2) and agonists selective for the A**_**2A**_ **AR (3, 4) and A**_**1**_ **AR (5).**

In 2011, the first crystal structure of the A_2A_AR bound to an agonist, i.e. the highly substituted nucleoside UK432,097 1 (Figure 1), was determined by Raymond Stevens and coworkers at Scripps Research Inst. (Xu et al. 2011), and similar structures of the A_2A_AR in complex with adenosine and its mono-substituted derivative (NECA, 2) were determined by Lebon et al. at MRC Laboratory of Molecular Biology in Cambridge, UK and Heptares Therapeutics (Lebon et al. 2011). The structures of Lebon et al., which additionally show portions of the second extracellular loop (EL2) not defined in the UK432,097 complex, were determined using receptors with thermostabilizing point mutations (StaRs). All agonist-bound A_2A_AR structures show similar interactions involving the adenine core and the ribose ring with the receptor. Moreover, the UK432,097 complex is stabilized by multiple H-bonding, van der Waals and hydrophobic interactions on extended C2 and N^6^ substituents. The agonist interactions within the A_2A_AR evident in the X-ray structures are shown below:

Comparison of agonist-bound and inactive conformations of the A_2A_AR have largely validated previous approaches to homology modeling of ARs and indicated the conformational changes needed for receptor activation, particularly within the ligand binding region. These conformational changes include a tightening of hydrophilic residues in TM3, TM5 and TM7 around the ribose moiety, which is known to form the “message” portion of the molecule (Xu et al. 2011). Many of the hydrogen bonds with ligand found in the agonist-bound A_2A_AR structures were predicted by earlier molecular modeling (Ivanov et al. 2009). Upon binding of nucleoside agonists to the ARs, unstable water molecules that appear in the inactive state of the A_2A_AR (Jaakola et al. 2008;Dore et al. 2011) are expelled from deep in the binding site. The more hydrophobic adenine moiety, which may be derivatized at the C2 and N^6^ positions with other mainly hydrophobic groups that extend further into the EL region, is considered the “address” portion of adenosine, with respect to receptor subtype selectivity. Some of the movements of ELs are specific to accommodate sterically bulky groups of UK432,097 and are less pronounced in other agonist-bound A_2A_AR structures. The implications for receptor signaling by particular agonists that produce their own distinct conformational changes of the A_2A_AR are not yet known.

Pharmacophore-based searches have yielded ligands having novel chemotypes, such as diverse heterocyclic antagonists of the A_2A_AR, a target for Parkinson’s disease (de Lera Ruiz et al. 2013). An inactive state structure of the A_2A_AR was applied to *in silico* searches of diverse chemical libraries to identify antagonists of several AR subtypes. This approach has been useful for discovery of antagonists at both the A_2A_AR (Katritch et al. 2010;Carlsson et al. 2010;van der Horst et al. 2011;Congreve et al. 2012;Langmead et al. 2012) and the closely related A_1_ and A_3_ARs, by homology. Four separate homology models of the human A_1_AR were built based on optimization with different bound (known) ligands of that subtype, and 2.2 million lead-like compounds were docked in all four models (Kolb et al. 2012). In this study, initially targeting the A_1_AR, 39 *in silico* hits were obtained and subsequently screened in binding to three AR subtypes, resulting in the following percent of successful antagonist hits identified: 21% (A_1_), 38% (A_2A_) and 36% (A_3_). Thus, this docking approach is useful for discovery of hits at the A_1_AR and at closely related subtypes. Some of the successful hits bound to two or three AR subtypes.

Beyond screenings for novel chemotypes, the structures of the A_2A_AR have also proved useful to design improved analogs of the existing ligands. Previous efforts to develop AR agonists for therapeutic use have faced efficacy issues and low selectivity, leading to side effects (Chen et al. 2013). Two agonists of the A_3_AR that were first synthesized and characterized biologically in our lab are already in advanced clinical trials for inflammatory diseases (IB-MECA, 3) and liver cancer (the 2-chloro analogue of IB-MECA, CF102). Current or planned trials of A_3_AR agonists are for treatment of hepatocellular cancer, rheumatoid arthritis, psoriasis, dry eye syndrome, and other inflammatory conditions. Moreover, selective A_1_AR agonists are sought as antiseizure agents, analgesics and for other neuroprotective functions (Luongo et al. 2013). Structural insights, enabled by molecular modeling, have guided the introduction of novel adenosine derivatives as A_1_ and A_3_AR agonists. A first step to validation of the AR agonist modeling approach was to apply it to the A_2A_AR, used as template, and then to extend it to the closely related A_1_ and A_3_AR by homology. Molecular modeling of agonist binding has been evaluated in light of the crystallographic structure of the agonist-bound A_2A_AR and found to predict interactions of 20 known nucleoside ligands (Deflorian et al. 2012).

**Figure 2:**
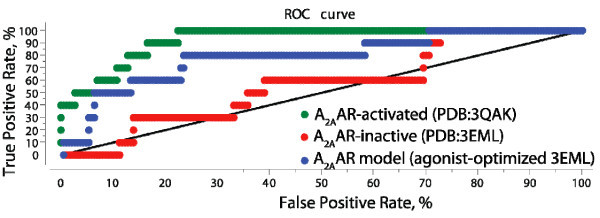
**Performance of virtual screening of amide fragment derivatives similar to NECA, using three different A**_**2A**_ **AR models, reprinted with permission from Tosh et al. (Tosh et al.** 2012c **).**

Ligand docking and fragment searching are useful for suggesting new congeners of known AR agonists and antagonists to be synthesized in order to explore distinct subpockets in the binding site. Our structure-based synthesis of adenosine agonists guided by knowledge of the X-ray structure treated each region of the nucleoside using a different approach. For example, binding affinities following distal changes on an extended C2 chain correlated with predicted interactions in the spatially permissive EL region (Deflorian et al. 2012). In contrast, an exploration of the relatively small subpocket of the agonist-bound A_2A_AR structure surrounding the 5′ substituent of ribose yielded novel 5′-amide derivatives, similar to NECA (Tosh et al. 2012c). The ethyl group of NECA was replaced with small (MW < 150) but diverse molecular fragments to complement this subpocket. The majority of *in silico* hits that were high ranking energetically (roughly the top 1% of a set of 2000 screened structures) and tested in binding assays indeed bound with submicomolar K_i_ values at the A_2A_AR. When the inactive A_2A_AR structure (3EML) was used for screening these adenosine analogues with diverse 5′-amide fragments, the statistical advantage shown in the receiver operating characteristic (ROC) curve (Figure 2), clearly evident using the agonist-bound A_2A_AR structure (3QAK), disappeared. When an intermediate conformational model (agonist-optimized 3EML) was used for screening, modified from the inactive state through docking of an agonist and relaxing of side chains, the ROC predictability was also intermediate. Virtual hits that more potently inhibited radioligand binding at the A_1_AR than the A_2A_AR were rationalized based on specific H-bonding interactions with residues of the A_1_AR as predicted in the modeling.

A comparison of the ribose binding elements of the A_2A_AR with the corresponding amino acid residues in other subtypes shows more significant variation in the A_3_AR than in the A_1_AR. Affinity (but not efficacy in receptor activation) of nucleosides at the A_3_AR seems to depend more heavily on factors outside the ribose binding pocket than is the case for other AR subtypes. This hypothesis is supported by many adenosine analogues lacking the 5′-amide group entirely, i.e. truncated at the 4′ carbon that still bind potently to the A_3_AR (Tosh et al. 2012b). Nevertheless, the tetrahydrofuryl core scaffold of ribose, or an isostere thereof, is still important at the A_3_AR as for other AR subtypes.

The latest generation of A_3_AR receptor agonists contains a constrained bicyclic (bicyclo[3.1.0]hexane, known as methanocarba) substitution of ribose to maintain a receptor-preferred North (N) conformation (Tosh et al. 2012a). This rigid ring system (fused cyclpentane and cyclopropane rings) in its two isomeric forms, i.e. either (N) or South (S), as applied to nucleosides was used before any structural elucidation to interrogate the ribose conformation when bound to an AR (as ribose in adenosine can readily twist to adopt a range of conformations) (Jacobson et al. 2000). The adenosine analogue having a fixed (N) conformation was clearly the more potent isomer at the A_1_AR, A_2A_AR and A_3_AR, with the greatest enhancement over (S) occurring at A_3_AR (155-fold). Indeed, the predicted (N) ribose conformation was verified when the agonist-bound A_2A_AR structures were determined.

We have explored rigidified (N)-methanocarba C2-arylalkynyl nucleoside analogues, such as MRS5698 4, that are highly selective A_3_AR agonists. The very high A_3_AR selectivity (typically ~10,000-fold) in this series of C2-arylalkynyl (N)-methanocarba nucleosides was consistent with a predicted outward movement of the portion of TM2 near the EL region of the A_3_AR, in order to preserve hydrogen-bonding interactions with conserved residues in TMs 3, 5, and 7 that lock the adenosine moiety in its deeper binding pocket. This outward movement of TM2, which is justified based on other activated GPCR structures (opsin and β_2_-adrenergic receptor) is not likely to occur in the A_2A_AR, because the EL region of this AR subtype is conformationally constrained by four disulfide bridges.

A_1_AR agonist MRS5474 5 also contains a constrained bicyclic substitution of ribose to maintain a receptor-preferred North (N) conformation (Tosh et al. 2012b). MRS5474 has in vivo anti-convulsant activity without typical rotorod toxicity observed for standard A_1_AR agonists, which are known to produce intense cardiovascular depression. The *N*^6^-dicyclopropylmethyl substitution was selected by screening (in binding assays of the synthetic analogues), in the truncated, methanocarba series, many alkyl/aryl groups known to promote A_1_AR agonism in the ribose series (Tosh et al. 2012b). This particular N^6^ group appears to be a structural sweet spot, in that it maintains selectivity and full efficacy. The very tight fit in the N^6^ region in receptor docking has been analyzed through homology modeling of the human A_1_AR, based on the agonist-bound A_2A_AR structure (Xu et al. 2011). Deletion of an otherwise important recognition element (H-bonding groups at the 5′ position) is compensated by a specific N^6^ substitution in MRS5474.

Novel fluorescent and radiolabeled probes and multivalent conjugates of strategically functionalized AR ligands have also been designed with the aid of molecular modeling (Kozma et al. 2013). Such high affinity ligands containing reporter groups are useful tools for the characterization of receptors and their oligomeric assemblies. For example, adenosine agonist binding to the heterodimers formed between the A_2A_AR and D_2_ dopamine receptor can be followed in real time using such a conjugate (Fernández-Dueñas et al. 2012). The interaction of the fluorophore of a given fluorescent chemical probe with the outer portions of the receptor, mainly distal interactions with functional groups on the receptor, may have a major influence on its affinity and selectivity. Thus, the pharmacological profile of the functionalized congener that serves as precursor of the fluorescent ligand is not always predictive of the properties of the final conjugate, and GPCR molecular modeling can provide insight into this phenomenon. One such example is a fluorescent agonist of the P2Y_6_R that was greatly enhanced in potency and selectivity by the addition of an AlexaFluor488 moiety at the end of a flexible chain (Jayasekara et al. 2013). According to receptor modeling, this fluorophore did not escape the outer environment, i.e. the ELs of the P2Y_6_R where they are predicted to anchor to specific sites. In this case, the closer template for homology modeling for the P2YRs was judged to be the CXCR4 structure, rather than the A_2A_AR structure.

## Conclusions

Thus, the 3D knowledge of receptor binding and activation promises to enable drug discovery for GPCRs in general, and specifically for the ARs. The predictability of modeling based on the X-ray structures of the A_2A_AR has been well demonstrated in the design and modification of both known and novel AR agonists and antagonists. *In silico* screening by docking of chemical libraries to these new structures and to closely related receptor homology models has been validated. It is expected that structure-based design of drugs acting through ARs will provide new avenues to clinically useful agents.
